# CD8^+^ T Cell-Mediated Neuronal Dysfunction and Degeneration in Limbic Encephalitis

**DOI:** 10.3389/fneur.2015.00163

**Published:** 2015-07-15

**Authors:** Petra Ehling, Nico Melzer, Thomas Budde, Sven G. Meuth

**Affiliations:** ^1^Department of Neurology, Westfälische Wilhelms-University of Münster, Münster, Germany; ^2^Institute of Physiology I – Neuropathophysiology, Westfälische Wilhelms-University, Münster, Germany; ^3^Institute of Physiology I, Westfälische Wilhelms-University, Münster, Germany

**Keywords:** limbic encephalitis, cytotoxic CD8^+^ T cell, T cell–neuron interaction, future strategies, autoimmune neurological disease

## Abstract

Autoimmune inflammation of the limbic gray matter structures of the human brain has recently been identified as major cause of mesial temporal lobe epilepsy with interictal temporal epileptiform activity and slowing of the electroencephalogram, progressive memory disturbances, as well as a variety of other behavioral, emotional, and cognitive changes. Magnetic resonance imaging exhibits volume and signal changes of the amygdala and hippocampus, and specific anti-neuronal antibodies binding to either intracellular or plasma membrane neuronal antigens can be detected in serum and cerebrospinal fluid. While effects of plasma cell-derived antibodies on neuronal function and integrity are increasingly becoming characterized, potentially contributing effects of T cell-mediated immune mechanisms remain poorly understood. CD8^+^ T cells are known to directly interact with major histocompatibility complex class I-expressing neurons in an antigen-specific manner. Here, we summarize current knowledge on how such direct CD8^+^ T cell–neuron interactions may impact neuronal excitability, plasticity, and integrity on a single cell and network level and provide an overview on methods to further corroborate the *in vivo* relevance of these mechanisms mainly obtained from *in vitro* studies.

## Role of Neuronal Antigen-Reactive CD8^+^ T Cells in Limbic Encephalitis – The Story So Far

### Clinical features of limbic encephalitis

Patients with limbic encephalitis (LE) (1–3) usually present with new onset mesial temporal lobe seizures, progressive memory disturbance, and a variety of other behavioral, emotional, and cognitive changes. Cerebrospinal fluid (CSF) exhibits inflammatory changes including lymphocytic pleocytosis, elevated protein, as well as intrathecal immunoglobulin (Ig)G synthesis or oligoclonal bands (OCB). The electroencephalogram (EEG) typically shows temporal epileptiform activity and slowing. Magnetic resonance imaging (MRI) exhibits volume and signal changes of the amygdala and hippocampus suggesting a sequence of acute inflammation followed by inflammation-driven neurodegeneration (4–7). Moreover, serum and CSF may contain specific auto-antibodies binding to either intracellular or plasma membrane-bound neuronal antigens (8–10) illustrating the presence of an adaptive neuron-directed autoimmune reaction.

### Putative immunopathogenesis of limbic encephalitis

Antigen-specific cellular and humoral immune responses directed toward central nervous system (CNS) neurons are believed to develop as a multi-step process (8, 11, 12). Soluble or cell-bound neuronal antigens or epitopes resembling them are engulfed and presented in the context of major histocompatibility complex (MHC) II molecules to CD4^+^ T cells by professional antigen-presenting cells (APCs) within secondary lymphatic organs (e.g., cervical lymph nodes). This in turn permits CD4^+^ T cells to license APCs to cross-present these antigens in the context of MHC I molecules to naïve CD8^+^ T cells, which then become activated and acquire cytotoxic effector functions (cellular effectors). Moreover, naïve B cells, which encounter, ingest, and present their cognate antigen in the context of MHC II molecules to CD4^+^ T cells, are in turn activated and become antibody-secreting plasma cells (humoral effectors). Following peripheral activation, both antibody-secreting plasma cells and cytotoxic CD8^+^ T cells (together with CD4^+^ T cells) may enter the CNS to attack neurons and cause functional and structural impairment (9, 10, 13). In general, both effector arms of the adaptive immune response may be activated irrespective of the cellular localization of the neuronal antigen or its antigenic epitope (plasma membrane vs. interior cellular compartments).

In terms of relevant effector mechanisms, plasma cell-derived antibodies bind to extracellular conformational epitopes of neuronal plasma membrane antigens and specifically impact their function, expression, and localization. Whether antibodies may also bind to and impact the function of intracellular neuronal antigens either by passive uptake into the neuron or by active binding to intracellular antigens, which are transiently exposed to the plasma membrane, is currently a matter of debate (14–17). In contrast, cytotoxic CD8^+^ T cells usually recognize linear peptides derived from antigens located in interior cell compartments following their MHC I-bound presentation on the neuronal surface (13). Whether peptides derived from neuronal surface membrane antigens are also presented to cytotoxic CD8^+^ T cells in the context of MHC I molecules is unclear at present (12).

Indeed, several findings suggest a pathogenic role of cytotoxic CD8^+^ T cells for neuronal damage in different forms of LE are as follows (18, 19): (i) neuronal damage often correlates with the number of CD8^+^ T cells, (ii) CD8^+^ T cells are found in close spatial proximity to neurons within the CNS, (iii) CD8^+^ T cells show an activated phenotype with substantial expression of the effector molecules (e.g., perforin and granzymes) in cytotoxic granules with a polar orientation toward neuronal cell membranes, (iv) some CD8^+^ T cells stain positive for CD107 indicating recent exocytosis of cytotoxic granules (i.e., degranulation), (v) neurons exhibit substantial cell surface expression of MHC I molecules allowing for cognate antigen-recognition by CD8^+^ T cells, and (vi) CD8^+^ T cells exhibit a restricted T cell receptor (TCR) repertoire (i.e., oligoclonal expansions), suggesting that they have expanded from a few precursors locally responding to distinct antigen epitopes in the CNS.

**Figure 1:**
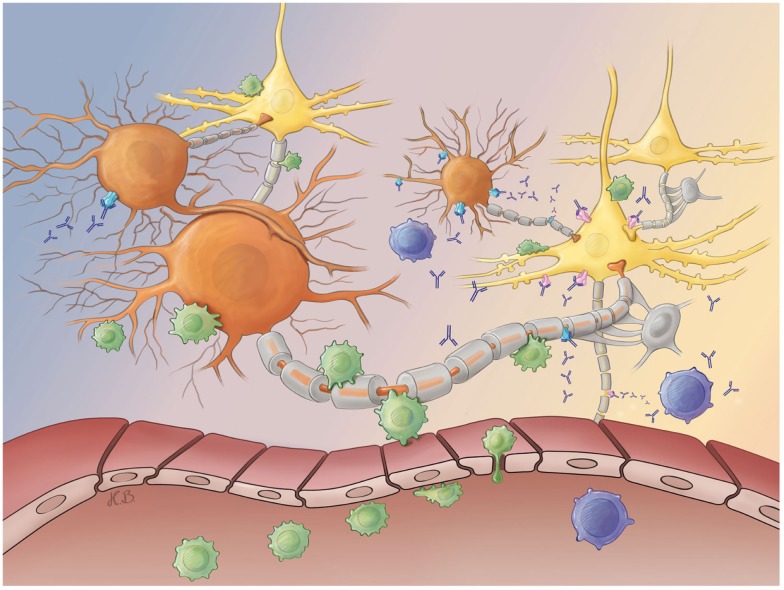
**Antibody- and T cell-mediated neurotoxicity**. After their peripheral activation, antibody-releasing plasma (blue) and activated CD8^+^ T cells (green) cross the blood–brain barrier and migrate into the brain parenchyma. Both effector arms of the adaptive immune system may selectively attack either inhibitory GABAergic interneurons (orange) or excitatory glutamatergic principal neurons (yellow) at synaptic as well as extra-synaptic sites. While plasma cell-derived antibodies may bind to neuronal surface antigens [e.g., GABA receptors (blue) or glutamate receptors (pink)], pathogenic CD8^+^ T cells recognize peptides derived from intracellular antigens (e.g., GAD in inhibitory interneurons or Hu in excitatory principal neurons) presented in the context of a MHC I molecule on the neuronal surface membrane. Both antibody- and T cell-mediated attacks finally cause neuronal dysfunction and degeneration. Abbreviations: GABA, γ-amino butyric acid; GAD, glutamic acid decarboxylase; MHC, major histocompatibility complex.

Hence, ongoing studies focus on the hypothesis that the encephalitides with antibodies against intracellular antigens [e.g., glutamic acid decarboxylase (GAD)] show neurodegeneration mediated by T cells, while encephalitides with antibodies against surface antigens [e.g., γ-amino butyric acid (GABA) receptor] are antibody mediated (Figure 1) (18, 20). These findings indicate that substances increasing GABA signaling may prevent tissue damage and seizures (21).

In contrast to antibodies, CD8^+^ T cells cannot directly impact the function or expression of their cognate antigens. Instead, cytotoxic T cells recognize their specific antigen only when presented on MHC I molecules on the surface of respective neuronal subtypes. This enables them to contribute to neuronal dysfunction and cell death by antigen-dependent release of effector molecules (13), as discussed below. Mouse data suggest that neuronal vulnerability against CD8^+^ T cell attacks might vary owing to their propensity to upregulate MHC class I molecules in response to inflammatory IFN-γ release (22). Moreover, catecholaminergic neurons in mice and humans have been shown to be particularly susceptible to T cell-mediated cytotoxic attacks (23). Thus, cellular immune responses may be restricted to distinct neuronal populations and networks due to their differential antigen expression pattern and capability of MHC I-mediated antigen presentation (22, 23). Moreover, distinct neuronal populations may take up soluble protein antigens released from other neural cell populations, process and present them in the context of MHC I molecules, thereby triggering a CD8^+^ T cell attack (23).

### Effector mechanisms during CD8^+^ T cell–neuron interactions *In vitro*

It has generally been presumed that the CNS is an immune-privileged organ, and that MHC I molecules are not expressed on neurons (24). However, CD8^+^ T cell-derived IFN-γ has recently been shown to cause immediate loss of dendrites and synapses, i.e., deafferentiation of neurons followed by delayed loss of neuronal somata in CNS gray matter areas (25). Reduced synaptic input through neuronal deafferentiation together with proinflammatory cytokine release may, in turn, reduce neuronal electrical activity below a critical threshold. Consequently, MHC I expression (22, 23) and endogenous or even exogenous antigen presentation are promoted and may thus render neurons susceptible for an antigen-dependent CD8^+^ T cell attack (26, 27). Consistently, it has recently been shown that MHC I expression *per se* in turn exerts profound effects on neuronal long-term plasticity in mice (28–33).

After encountering such neurons that present cognate antigens in the context of MHC I molecules, CD8^+^ T cells arrest and undergo stable long-term interactions (13, 34). TCR-signaling upon recognition of the appropriate antigen in the context of MHC I molecules leads to redistribution and accumulation of cytoskeletal, adhesion, co-stimulatory, and signal transduction molecules of the CD8^+^ T cell toward the cell–cell interface, resulting in the formation of the immunological synapse (18, 35). Similar to those formed by CD4^+^ T cells, the synapses formed by cytotoxic T cells during killing of their target consists of a ring of adhesion proteins surrounding a central core containing the TCR and downstream signaling proteins. However, synapses in CD8^+^ cells additionally possess a secretory domain for the exocytosis of effector molecules and reveal a shorter lifespan compared to CD4^+^ cell synapses (35).

CD8^+^ T cell-mediated cytotoxicity is predominantly mediated via two largely independent pathways (36, 37): (i) Granule cytotoxicity occurs by release of perforin together with a variety of granzymes. Perforin alone can lead to rapid necrosis of the target cell within minutes through the formation of large unselective transmembrane pores leading to rapid swelling and rupture of the cell membrane (38). Alternatively, perforin mediates the trafficking of granzymes into the target cell promoting apoptosis within a few hours. The exact mechanisms remain somewhat elusive (38, 39). (ii) Target cell apoptosis may also occur through the ligation of cell death receptors [e.g., FasL/Fas; (40)]. Together, Fas-induced apoptosis and the perforin pathway are the two main mechanisms by which cytotoxic T lymphocytes induce cell death in cells expressing foreign antigens (41).

The use of either the FasL–Fas or the perforin–granzyme pathway of CD8^+^ T cells depends on the strength of the antigen-signal delivered to the CD8^+^ T cell [i.e., the number of peptide (p) MHC I (pMHC I) complexes and the affinity of the TCR complex including co-receptors to the pMHC I complex]. This eventually results in different intracellular Ca^2+^ signals in T cells. Weak antigen-signals favor killing via the FasL–Fas pathway, whereas strong antigen-signals promote killing via perforin–granzyme exocytosis (42–44). Notably, 1–3 pMHC I-complexes per neuron are shown to be sufficient to elicit a cytotoxic T cell response when the TCR–pMHC I-affinity is high (44, 45). However, in case of low TCR–pMHC I-affinity, several thousand pMHC I complexes per target cell are needed to elicit an equal response (46).

### Impact of CD8^+^ T cells on neuronal excitability and neuronal network activity

Besides the induction of cell death, effector molecules of cytotoxic CD8^+^ T cells are capable of disturbing electrical signaling in excitable target cells. The impact of these molecules on the electrical excitability has been extensively studied in ventricular cardiomyocytes but not neurons (47, 48).

Within minutes, purified perforin or lytic granules exposed to ventricular cardiomyocytes cause membrane depolarization as well as changes in amplitude and duration of action potentials. These effects are mediated by perforin *per se* and cannot be induced by granzymes alone (49, 50). Perforin monomers assemble to form large, unselective voltage-independent polyperforin channels in the target cell membrane (49, 50). This allows large non-selective ion fluxes over the plasma membrane, as also shown in lipid bilayer membranes and other intact cells (51, 52). After 2 h, affected cells exhibit an intracellular Ca^2+^ concentration in the micromolar range compared to low-nanomolar concentrations under physiological resting conditions. This is most likely due to transmembrane Ca^2+^ entry through perforin pores rather than through voltage-gated Ca^2+^ channels or by Ca^2+^ release from intracellular stores (53). Most importantly, these ion fluxes lead to the abolishment of transmembrane electrochemical ion gradients, an intracellular Ca^2+^ overload, and finally result in total electrical silence and collapse of the target cell.

Similarly, exposition with activating anti-Fas-receptor antibodies as well as conjugation with perforin-deficient CD8^+^ T cells also induced pronounced perturbation of electrical signaling in ventricular cardiomyocytes. The cells’ resting membrane potentials depolarize and their action potential amplitudes are reduced. Notably, the action potential duration is prolonged, a finding in marked contrast to the effects of perforin (54). Fas-receptor activation results in generation of 1,4,5-inositol-trisphosphate (IP_3_), which in turn triggers the release of Ca^2+^ from intracellular stores. Fas activation further causes a pronounced attenuation of transient outward K^+^ currents and an enhancement of L-type Ca^2+^ currents, and thus, prolongs the action potential duration. Electrophysiological effects of Fas-receptor activation can be mimicked by intracellular application of IP_3_ and can be abrogated by blocking phospholipase C, the IP_3_ receptor channel or store depletion (47, 54). Together, similar to perforin, Fas activation results in an intracellular Ca^2+^ overload of ventricular cardiomyocytes within a few hours.

In neurons, Ca^2+^ overload is associated with long-lasting changes in neuronal Ca^2+^ homeostasis and disturbed functioning of several Ca^2+^-dependent proteins (55–58). Furthermore, provided that most of the subcellular elements required for CD8^+^ T cell-mediated impairment of electrical excitability in cardiomyocytes are also present in neurons (59), it seems conceivable to assume that similar mechanisms will also lead to perturbation of neuronal excitability and Ca^2+^ homeostasis upon direct and indirect CD8^+^ T cell-neuron interactions.

In a previous study from our group, we used whole-cell patch clamp recordings and Ca^2+^ imaging from MHC I-expressing, ovalbumin (OVA)–peptide loaded cultured hippocampal neurons (60). Thereby, we demonstrated an immediate increase of the whole-cell membrane conductance upon direct cell–cell contact with activated antigen-specific CD8^+^ T cells leading to an impairment of electrical signaling (silencing). This was due to shunting of the membrane capacitance following insertion of CD8^+^ T cell-derived channel-forming perforin, which was paralleled by an increase of intracellular Ca^2+^ levels. Thus, perforin-dependent neuronal silencing is an immediate consequence of MHC I-restricted interaction of CD8^+^ T cells with cultured neurons much like the effects observed in cardiomyocytes (13, 60).

Importantly, an increase of the intracellular Ca^2+^ concentration could not only be detected in the neuron directly engaged with the CD8^+^ T cell but also in neighboring neurons without direct T cell contact (60). The nature of this “spill over-mechanism” explaining the intracellular Ca^2+^ accumulation in neighboring neurons has not been studied in detail. Possible mechanisms include that either membrane depolarization or Ca^2+^ accumulation causes intense synaptic signaling within the network of cultured neurons. However, glutamate toxicity is unlikely because glutamate could not be detected in culture supernatants after granule-induced cytotoxicity (61). Thus, these *in vitro* data indicate that only spatially confined *trans*-synaptic neuronal excitotoxicity involving activation of the respective ionotropic glutamate receptors might further promote neuronal damage. Alternatively, spatially non-confined release of cytotoxic effector molecules from the CD8^+^ T cell in contact with the neuron could also explain collateral effects in surrounding neurons (13).

Moreover, CD8^+^ (and CD4^+^) T cells have been shown to release glutamate that may contribute to effects on remote neurons within a distinct network (62, 63). Also, cytokines are released from CD8^+^ T cells and might affect the excitability and viability of neuronal networks. Thus, INF-γ has been shown to enhance glutamate excitotoxicity by direct intracellular *trans*-signaling between its INF-γ and AMPA/kainate receptors (64). TNF-α is shown to have an intrinsic ion channel-forming activity. Similar to polyperforin molecules, TNF-α trimers form largely unselective, high-conductance ion channels that may insert into lipid bilayer or cell membranes promoted under low pH values (65–67). Moreover, other inflammatory mediators are also likely to contribute to the perturbation of excitability and structure of neuronal networks [e.g., IFN-α and IL-1β in hypothalamic slice preparations, intraventricular IL-2 injections; reviewed in Ref. (68)].

These findings suggest a profound disturbance of neuronal function in close vicinity to as well as remote from the site of direct CD8^+^ T cell–neuron interaction and thus a significant impact of CD8^+^ T cells on structure and function of distinct neuronal networks (60). Significant limitations of this study, however, include that it was performed using neurons loaded with non-limiting amounts of exogenous OVA peptides following IFN-γ-induced MHC I expression. Moreover, CD8^+^ T cells had a transgenic TCR specific for the respective OVA–peptide and were strongly pre-activated in culture before experimentation.

These limitations have been partially overcome by the use of virus-infected neurons incubated with virus-specific CD8^+^ T cells isolated from brains of infected rodents (34). In this experimental setting, CD8^+^ T cells caused an immediate profound increase of neuronal network activity in multi-electrode recordings (34), suggesting that depending on the experimental conditions, direct CD8^+^ T cell–neuron interactions may effectively modulate electrical signaling within neuronal networks.

However, as most of these findings have been obtained from *in vitro* studies, the *in vivo* relevance of these results awaits demonstration.

### Lack of tools for the study of CD8^+^ T cell-neuron interactions and potential methods of resolution

Despite the clear evidence for a pathogenic role of CD8^+^ T cells in autoimmune inflammation of the limbic gray matter structures constituting LE and other conditions, it has been difficult to develop an adequate *in vivo* model of autoimmune CD8^+^ T cell-mediated CNS inflammation directed against an endogenous neuronal antigen in rodents. In adoptive transfer experiments with myelin-reactive encephalitogenic CD8^+^ T cells, analogous to those performed with encephalitogenic CD4^+^ T cells, it was extremely difficult to induce clinically apparent disease in rodents. For example, transfer of very high numbers (3 × 10^7^, corresponding to about 10% of the endogenous CD8^+^ T cell population) of *in vitro* pre-activated hemagglutinin (HA)-specific CD8^+^ T cells induces experimental autoimmune encephalitis (EAE) in less than half of recipient mice expressing HA as a neo-self antigen in oligodendrocytes (69). In another EAE-model employing TCR-transgenic myelin basic protein (MBP)-specific CD8^+^ T cells, disease induction requires transfer of 2 × 10^7^ pre-activated cells (70). In contrast, as few as 1 × 10^5^ MBP-specific CD4^+^ T cells suffice to induce EAE in mice (71), indicating that the pathogenic potency of activated CNS–antigen reactive CD4^+^ T cells exceeds that of their CD8^+^ counterparts by more than two orders of magnitude. Similarly, strong tolerance also exists for CD8^+^ T cell reactions toward endogenous neuronal antigens (72, 73). This is most likely to be distinct but poorly understood central and peripheral (74) as well as CNS-specific (75) tolerance mechanisms (13). These physiological mechanisms might establish a strict regulation of such immune responses given their strong destructive potential, especially in target organs with limited regenerative capacity, such as the CNS.

However, in case of distinct foreign neuronal antigens, CD8^+^ T cell–neuron interactions could be studied *in vivo* without requiring the break of strong self-tolerance to endogenous neuronal antigens (25, 34, 76).

Non-neuronal cells play a central role for the maintenance and functionality of neurons and networks in the CNS and may become targets of auto-reactive CNS-invading CD8^+^ T cells. For instance, in addition to neurons, astrocytes have been identified as targets for CD8^+^ T cells in Rasmussen’s encephalitis (RE) (77), a paradigmatic model of CD8^+^ T cell-driven neurodegeneration and epilepsy (78). Moreover, intracellular antigens expressed in oligodendrocytes have been described as targets of cerebral autoimmunity in CV2-antibody-associated encephalitis (79–81). In the context of several other inflammation-driven neurodegenerative diseases, it has already been shown that death of non-neuronal cells promotes subsequent neuronal loss [e.g., dying of oligodendrocytes promotes axonal degeneration in multiple sclerosis, Ref. (82)]. Further, it has been shown that cytotoxic effector CD8^+^ T cells selectively directed toward an oligodendrocyte-related neo-self-antigen are capable of inducing caspase-dependent apoptosis upon recognition of their cognate antigen, which is accompanied by simultaneous apoptosis of gray matter neurons (83, 84). Hence, impairment of neuronal structure and function may also occur as a collateral effect of an attack by CNS-invasive CD8^+^ T cells.

## Methodological Repertoire for the Study of Neuronal Effects of CD8^+^ T Cell Attacks: Possible Solutions to Pending Problems

Compared to other disease entities research in the field of LE pathogenesis is still in its early stages. Meanwhile, pathogenic effects of plasma cell-derived antibodies on neuronal function and integrity are increasingly becoming characterized (85–90), potentially contributing effects of T cell-mediated immune mechanisms are highly likely to be of pivotal importance but remain poorly understood.

In this respect, the following issues are of primary importance: (i) how do cytotoxic CD8^+^ T cells influence the functionality of neurons or neuronal networks?; (ii) it remains to be demonstrated that an attack of cytotoxic CD8^+^ T cells is a conclusive mechanism to provoke pathological hyperexcitability in a neuronal network that finally leads to temporal lobe epileptic seizures; and (iii) are non-neuronal cells affected by the immune attack in a way that involves collateral neuronal damage?

Very recently, a new mouse model for anti-NMDA encephalitis was described in which patient CSF (containing NMDA receptor specific auto-antibodies) was delivered directly into mouse brains via osmotic pumps thereby inducing behavioral and memory deficits in mice (86). However, the establishment of an animal model specifically reflecting LE-specific pathophysiology, namely, primary CNS-specific and CD8^+^ T cell-driven autoimmunity against neuronal targets, is still one of the most pressing tasks in LE research. Indeed, it has been demonstrated that several weeks following intrahippocampal kainate administration accompanied by acute symptomatic seizures, mainly CD8^+^ T cells accumulate in the parenchyma of lesioned hippocampi and seem to strongly modulate neuronal degeneration and reorganization leading to an attenuated neuronal network excitability and generation of spontaneous epileptic seizures implying a profound impact of secondary adaptive neuron-directed immunity on epileptogenesis in this model (91, 92). However, to date, no animal model exists, in which a primary CD8^+^ T cell response targets distinct neuronal populations based on an antigen-specific direct interaction. Such a disease model, however, would give insights into pathophysiological mechanisms *in vivo* and *ex vivo* as well as it would be beneficial for any kind of treatment studies addressing primarily immune-mediated epilepsies. Despite the lack of an adequate LE animal model, antigen-directed CD8^+^ T cell-mediated neuronal attacks can be mimicked in different experimental settings. For instance, *in vitro* activated MHC I-restricted, OVA-reactive T cells from OT-I mice (93) can be used in combination with neurons/neuronal tissue from NSE-OVA mice expressing OVA as a neuronal auto-antigen under control of the neuron-specific enolase promoter (94).

Our group published one of the first *in vitro* studies focusing on immediate electrophysiological consequences on a single neuron upon direct antigen-directed CD8^+^ T cell attack. Real-time patch clamp recordings from OVA-loaded cultured hippocampal neurons (s. above) were performed during an attack from an OVA-directed activated CD8^+^ T cell. Analyses of different basic electrophysiological parameters showed that the cytotoxic attack against OVA-presenting neurons led to cellular shrinking and significant reduction of the membrane resistance compared to recordings with control peptide. Increased neuronal membrane conductance during antigen-directed T cell–neuron interaction was attributable to incorporation of perforin into the neuronal surface membrane. Impairment of electrical signaling paralleled by Ca^2+^ overload occurred within minutes. However, antigen-dependent apoptosis was not necessarily dependent on perforin and granzymes indicating other, so far unknown CD8^+^ T cell-mediated mechanisms promoting cell death (60).

Besides our sparse knowledge about consequences on single neuron functions due to direct immune cell attacks, we can only speculate on changes on the network level. At this point, an early imaging technique, whose widespread usage has been limited due to the sophisticated technical effort necessary to run successful experiments (95), may prove useful. It makes use of living brain slices loaded with voltage-sensitive dyes that change their fluorescent properties upon alterations of the membrane potential. Upon a defined depolarizing stimulus, the strength and the spreading of a network response can be visualized with high-temporal–spatial resolution. Conclusions can be drawn about the network’s functionality and intra-connectivity (96). An alternative *in vitro* technique to record network activity uses multi-electrode arrays, which offer the capability of recording and stimulating at multiple sites simultaneously (97). This technique has already been used to investigate pharmacologically induced epileptiform network activity (98). However, to obtain insights into immune attack-induced changes of neuronal functioning *in vivo*, electrophysiological recordings from freely behaving animals need to be done. This advanced technique permits the observation of cross-talk between single or multiple neurons in interconnected brain areas in combination with different behavioral contexts [e.g., Ref. (99)].

Further classical electrophysiological techniques may be suitable and informative. Long-term plasticity measurements in living hippocampal slices from rodents could reveal direct neuronal effects of a cellular immune response on synaptic plasticity as a basis for learning and memory processes. Especially, since memory disturbances are one of the prominent LE symptoms, this technique can be used to approach this aspect of the disease.

## Conclusion

In recent years, an ever increasing number of endogenous neuronal plasma membrane auto-antigens have been identified as targets of specific auto-antibodies in LE and other autoimmune encephalitides. Pathogenic effects of auto-antibodies on neuronal excitability and integrity are increasingly becoming recognized on the single cell, network, and systems level. Auto-reactive T cells have been suggested to target neurons in autoimmune encephalitides associated with antibodies to endogenous intracellular neuronal antigens. However, the consequences of such direct T cell–neuron interactions remain poorly understood.

CD8^+^ T cell-derived IFN-γ may first contribute to neuronal MHC I up-regulation and antigen presentation. By a variety of molecular mechanisms, neuron-directed CD8^+^ T cell attacks may immediately modulate neuronal excitability and network activity over a wide range of functional states and trigger acute symptomatic seizures as well as neuropsychiatric symptoms. Subsequent inflammation-driven neuronal degeneration together with thus far poorly defined processes of neuronal reorganization may permanently alter structure and excitability in distinct neuronal networks targeted due to their specific antigen expression pattern and thus promoting chronic spontaneous seizures and epilepsy together with other neuropsychiatric symptoms. To corroborate these largely speculative mechanisms, effects of T cell–neuron interactions need to be studied on single cell, network, and systems level ideally in a suitable animal model using advance electrophysiological and imaging methods.

## Conflict of Interest Statement

All authors declare no relevant conflicts of interest. Petra Ehling has received honoria for lecturing, travel expenses for attending meetings, and financial research support from Merck Serono and Novartis. Nico Melzer has received honoria for lecturing and travel expenses for attending meetings from Biogen Idec, GlaxoSmith Kline, Teva, and Fresenius Medical Care and has received financial research support from Fresenius Medical Care. Thomas Budde has received financial research support from Bayer, Biogen Idec, and Novartis. Sven G. Meuth has received honoraria for lecturing and travel expenses for attending meetings and has received financial research support from Bayer, Bayer Schering, Biogen Idec, Genzyme, Merck Serono, MSD, Novartis, Novo Nordisk, Sanofi-Aventis, and Teva.
